# Metabolic reprogramming in colorectal cancer: regulatory networks and therapy

**DOI:** 10.1186/s13578-023-00977-w

**Published:** 2023-02-08

**Authors:** Jieping Zhang, Shaomin Zou, Lekun Fang

**Affiliations:** 1 Department of General Surgery, Guangdong Provincial Key Laboratory of Colorectal and Pelvic Floor Disease, The Sixth Affiliated Hospital, Sun Yat-Sen University, 26 Yuanchun Er Heng Road, Guangzhou, 510655 Guangdong China; 2 Guangdong Institute of Gastroenterology, Guangzhou, 510655 China

**Keywords:** Colorectal cancer, Metabolic reprogramming, Metabolic enzymes, Signal transduction, Targeted therapy

## Abstract

With high prevalence and mortality, together with metabolic reprogramming, colorectal cancer is a leading cause of cancer-related death. Metabolic reprogramming gives tumors the capacity for long-term cell proliferation, making it a distinguishing feature of cancer. Energy and intermediate metabolites produced by metabolic reprogramming fuel the rapid growth of cancer cells. Aberrant metabolic enzyme-mediated tumor metabolism is regulated at multiple levels. Notably, tumor metabolism is affected by nutrient levels, cell interactions, and transcriptional and posttranscriptional regulation. Understanding the crosstalk between metabolic enzymes and colorectal carcinogenesis factors is particularly important to advance research for targeted cancer therapy strategies via the investigation into the aberrant regulation of metabolic pathways. Hence, the abnormal roles and regulation of metabolic enzymes in recent years are reviewed in this paper, which provides an overview of targeted inhibitors for targeting metabolic enzymes in colorectal cancer that have been identified through tumor research or clinical trials.

## Introduction

CRC (colorectal cancer) is a leading cause of cancer-associated mortality worldwide, with more than 150,000 new cases and 50,000 deaths worldwide every year [1, 2]. CRC is a result of genetic, environmental, and lifestyle risk factors. Genetic dysregulation of metabolic enzymes is involved in the development and progression of CRC. Notably, genetic mutants (APC, KRAS, TP53, MYC, and SMAD4 mutants) in CRC have been reported to participate in genetically induced global metabolic reprogramming by regulating metabolic enzymes [3–9]. In addition to genetic events, cancer cells adapt to environmental stresses by acquiring specific metabolic properties. For example, an elevated lipogenesis rate is observed under hypoxic conditions [10]. Therefore, different types of regulatory networks in CRC cells integrate the transformation of metabolic flux to determine tumor cell fate. In this regard, the essential role of tumor cell metabolism has been confirmed via cancer therapy, revealing the vulnerability of these cells to inhibitors of metabolic enzymes.

Metabolic reprogramming is a hallmark of malignancy, and tumors display increased absorption and processing of nutrients to meet the demands of rapid proliferation [11]. The Warburg effect, first described by Otto Warburg in the 1920s, refers to the higher rate of tumor cells' conversion of glucose to lactate, even under aerobic conditions, than that of normal cells. Warburg’s pioneering efforts on aerobic glycolysis laid the foundation for the study of tumor metabolism [12]. Studies have since revealed that glycolysis produces ATP (adenosine triphosphate) faster than that produced by the TCA (trichloroacetic acid) pathway, which indicates the important role of glycolysis in supplying energy [13]. The reprogramming of glucose metabolism allows the diversion of glycolytic intermediates into various biosynthetic pathways, including pathway-generating lipids, nucleosides, and amino acids; these alterations facilitate, in turn, the biosynthesis of the macromolecules and organelles required for assembling new cells [14]. With a greater understanding of the complexity of tumor metabolism and biology, we have increased awareness of the significant metabolic heterogeneity between tumor and normal tissues, as well as within tumor tissues, and the metabolic characteristics and preferences that are constantly changing during tumor progression [15].

In this review, we briefly revisit important discoveries and concepts in the regulation of enzymes that are involved in cellular metabolism. Additionally, we describe several inhibitors that are currently being developed or used to target metabolic enzymes in CRC. In summary, we thoroughly characterize how metabolic enzymes interact with carcinogenic factors, which may help expand the current understanding of their potential roles in intestinal health and disease.

## Glucose metabolism in CRC

**Fig. 1:**
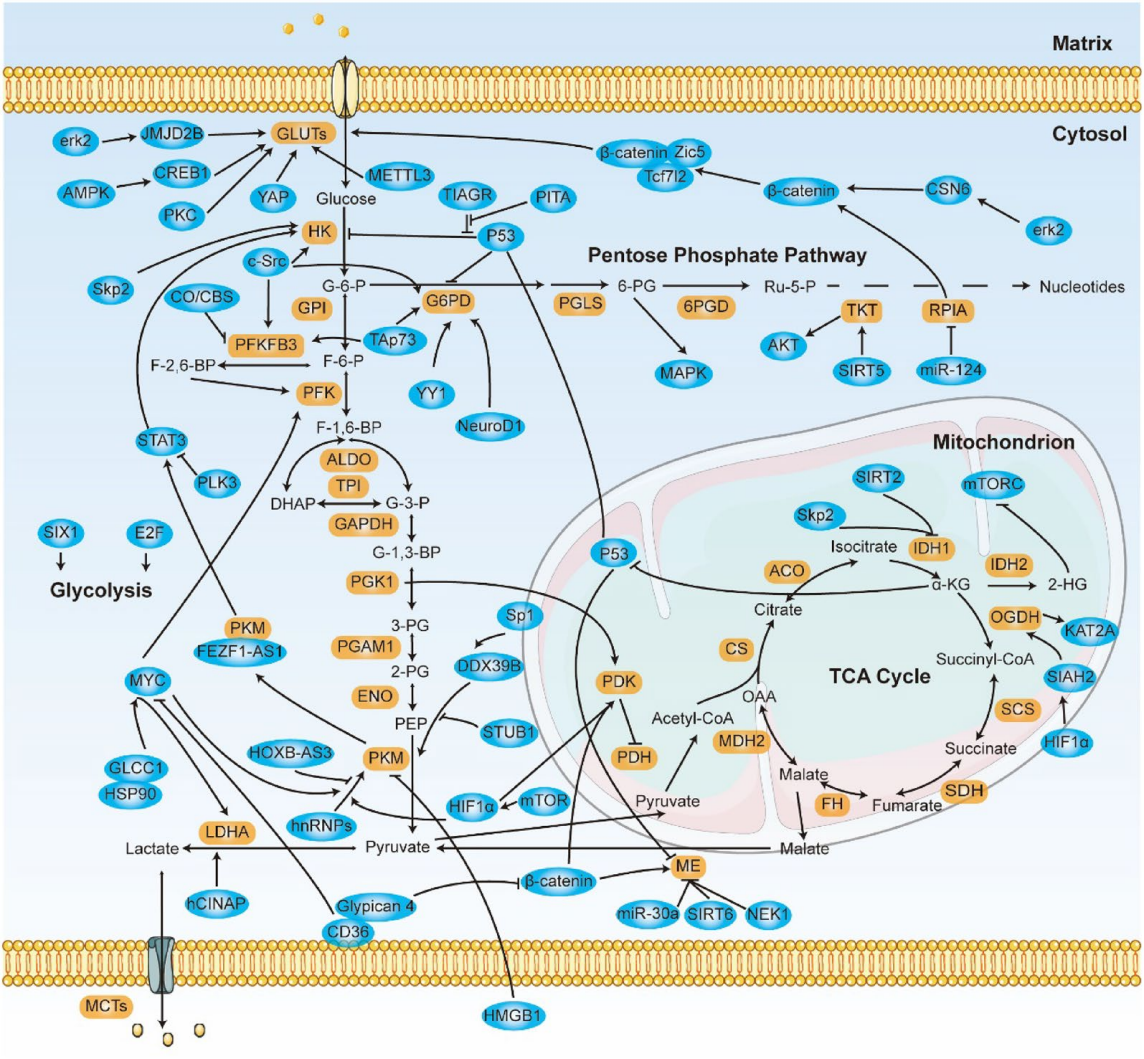
Schematic of Glucose Metabolism with Metabolic Enzymes and Their Regulators in CRC Cells. Glucose metabolic pathways mainly consist of glycolysis, the PPP pathway, and the TCA cycle; Enzymes involved in the glucose metabolism process moonlighting functions and are adapted for survival or death; are shown. Brown boxes indicate metabolic enzymes. Blue boxes represent factors that alter glucose metabolism

Glucose is the primary source of energy and supports important metabolic intermediates in cells, and this contribution of glucose is thought to exert great effects on tumor cell metabolism. Normally, glucose is catabolized to pyruvate by glycolysis in the cytosol and then transported to mitochondria, where it is further catabolized in the TCA cycle and via OXPHOS (oxidative phosphorylation) (Fig. 1). OXPHOS deficiency was initially thought to drive aerobic glycolysis, but this view has changed; the cellular requirement for biomacromolecules is now recognized as the driver of aerobic glycolysis [12]. In addition, glucose metabolism via the PPP (pentose phosphate pathway) provides the greatest portion of the ribonucleotides and NADPH (nicotinamide adenine dinucleotide phosphate) molecules that the cell needs [16]. Hyperactivated glucose uptake and aerobic glycolysis are considered hallmarks of tumor cells, because they fulfill the energy demand for high rates of proliferation [17]. Studies have revealed that the expression or activity of glucose metabolism enzymes is commonly changed in CRC, and we discuss the major alterations and the regulatory mechanisms.

### Aerobic glycolysis

Glycolysis mediates the conversion of glucose into pyruvate and produces ATP and NADH (nicotinamide adenine dinucleotide) in cells. Glucose uptake and aerobic glycolysis rates are frequently increased in CRC because they provide advantages for tumor development [18]. The high glycolysis rate in tumor cells depends on key metabolic enzymes and substrates, which are processed through large and intricate regulatory networks. Oncogenic transcription factors E2F1, MYC, and SIX1, for example, play a direct role in regulating the Warburg effect [19, 20]. Taken together, studies related to the key steps in aberrant tumor cell glycolysis can help us to detect and treat cancer (Fig. 1).

GLUT (glucose transporter) is critical for the uptake of glucose and is related to CRC. Dysregulation of β-catenin signaling has been closely linked to glucose metabolism. Transducer β-catenin forms a complex with Tcf7l2 and Zic5 that directly regulates the expression of GLUT1, resulting in glucose metabolism reprogramming [21]. Additionally, β-catenin promotes CRC tumorigenesis via the deregulation of β-catenin by ERK2 (extracellular regulated kinase 2)-activated CSN6 (COP9 signalosome subunit 6) [22]. PKC (protein kinase C)-mediated GLUT1 S226 phosphorylation regulates the rate of glucose transport and maintains the optimal level of membrane-stored GLUT1 [23]. CRC cells with *KRAS*^G12D, G13D^, and *BARF*^V600E^ mutations show upregulated expression of GLUT1 protein, which increases the absorption of dehydroascorbate, resulting in a high level of ROS (reactive oxygen species), which subsequently inactivates GAPDH (glyceraldehyde-3-phosphate dehydrogenase) [24]. The RNA N6-methyladenosine modification is linked to glycolysis via the methyltransferase METTL3-mediated N-methyladenosine in GLUT1, which results in the activation of mTOR (mammalian target of rapamycin) signalling [25]. The Hippo pathway also regulates metabolic reprogramming. Elevated GLUT3 activates YAP (yes-associated protein)-dependent metastasis, and activation of YAP in turn upregulates GLUT3 and PKM2 expression. Meanwhile, enhanced PKM2 interacts with YAP and enhances GLUT3 expression to promote CRC progression [26]. Glucose starvation-induced p-ERK phosphorylates and interacts with the demethylase JMJD2B (jumonji domain-containing protein 2B) to prevent its ubiquitination-related degradation, thereby reducing the abundance of the repressive H3K9me3 levels on the GLUT1 promoter to increase GLUT1 expression and prevent cancer cell death[27]. Similarly, starvation-induced AMPK (adenine monophosphate-activated protein kinase) activates CREB1 (cAMP response element binding protein 1), which transcriptionally increases GLUT3-mediated glucose uptake [28].

Before glucose enters bioactive pools, HK (hexokinase) converts glucose into G6P (glucose-6-phosphate) [29]. TIGAR (TP53 inducible glycolysis and apoptosis regulator) redirects glucose flux from glycolysis to PPP by exerting fructose-2,6-bisphosphatase activity. Under hypoxic conditions, TIGAR is translocated to mitochondria where it forms a protein complex with HK2, triggering glucose metabolism and maintaining cell survival by reducing ROS levels [30, 31]. PITA (P53 inhibitor of TIGAR activation), a KRAB-type zinc-finger protein, accelerates glucose into glycolysis by competitively binding to P53 with TIGAR [18]. The competitive regulatory network of P53, TIGAR, and PITA is thought to contribute to the ability of P53-mediated glycolysis to facilitate cell adaptation to metabolic stress [18, 31]. Skp2 (S-phase kinase-associated protein 2) is an E3 ligase subunit that is involved in CRC tumor development by increasing HK2-mediated aerobic glycolysis [32]. HK2 also mediates crosstalk between glycolysis factors and tumor suppressor PLK3 (polo-like kinase 3). PLK3 interacts with HSP90 and promotes HSP90 degradation, decreasing the phosphorylation of STAT3 (signal transducer and activator of transcription 3) to transcriptionally downregulate HK2 expression [33]. As an oncogenic tyrosine kinase, c-Src has been reported to contribute to glucose metabolism reprogramming. Activated c-Src phosphorylates HK1 Y732 and HK2 Y686, which are both crucial for c-Src-induced tumorigenesis by activating HK1/2 [34].

PFK1 (phosphofructokinase 1) is involved in maintaining the homeostasis of fructose-2,6-bisphosphate, which can be allosterically activated by PFKFB (6-phosphofructo-2-kinase/fructose-2,6-bisphosphatase)-catalyzed production of fructose-2,6-bisphosphate. In CRC cells, oncogenic MYC induces metabolic reprogramming partly by upregulating PFK [6]. TAp73 is a structural homolog of P53 and confers a proliferative advantage to CRC cells, which can promote glycolysis by upregulating PFK1 [35]. In addition to phosphorylating HK, c-Src phosphorylates PFKFB3 at Y194, which induces PFKFB3 to produce fructose-2,6-bisphosphate and allosterically activates PFK1 [36]. Furthermore, gas-responsive CO/CBS (carbon monoxide/cystathionine β-synthase)-dependent methylation of PFKFB3 is involved in CRC development and chemotherapy [37]. Stress-induced CO production suppresses CBS activity, resulting in a decrease in methylated PFKFB3 (R131/134), which promotes PFKFB3 K142 polyubiquitination and ubiquitination-related degradation, thereby driving glucose into the PPP and increasing cellular NADPH levels [37].

PKM (pyruvate kinase M) regulates the rate-limiting step of glycolysis and maintains a complex regulatory network. PKM1 and PKM2 are splice variants encoded by one *PK* gene, and between them, PKM2 is the greater contributor to CRC tumorigenesis [38]. The lncRNA HOXB-AS3 peptide competitively binds to splicing factor hnRNP A1 and inhibits hnRNP A1-dependent PKM2 maturation, suppressing glucose metabolism [39]. Similarly, activated mTOR stimulates HIF1α (hypoxia-inducible factor 1 subunit α) to enhance PKM2 expression through its collaboration with c-Myc–hnRNP splicing regulators, which together are critical for regulating cell proliferation and maintaining metabolic homeostasis [40]. In contrast to HOXB-AS3, the lncRNA FEZF1-AS1 interacts with and stabilizes PKM2 to activate STAT3 signaling [41]. Sp1 increases DDX39B levels to maintain PKM2 enzyme activity, while E3 ligase STUB1-mediated PKM2 proteasome degradation reduces PKM2 activity [42]. PKM2 mainly forms highly active tetramers and dimers that show low activity. The HMGB1 protein secreted by natural killer cells allosterically inhibits tetrameric PKM2 formation to induce a metabolic profile that leads to tumor cell death [43]. During CRC liver metastasis, upregulated PKLR (liver and kidney pyruvate kinase) can noncanonically destroy PKM2 enzyme activity and deregulate glycolytic metabolism to drive the malignant progression [44].

LDHA (lactate dehydrogenase A) is closely linked with cancer development because it stores high levels of lactate. Sustained self-renewal of stem cells determines CRC progression. Studies have shown that the adenylate kinase hCINAP mediates the self-renewal of CRC stem cells by accelerating the phosphorylation of LDHA at Y10 [45]. The membrane glycoprotein CD36 inhibits aerobic glycolysis in CRC cells. CD36 conjuncts with Glypican 4 to form the GPC4 complex, which facilitates the proteasome-dependent ubiquitination of GPC4, further blocking β-catenin/c-Myc signaling and reducing the expression of downstream glycolytic target genes [46]. In addition, in combination with HSP90, the lncRNA GLCC1 stabilizes MYC protein by protecting it from ubiquitination-related degradation and subsequently regulates LDHA to reprogram glycolytic metabolism [47]. Intriguingly, lactate has also been reported to promote tumor development. In CRC stem cells under normoxic conditions, tumor microenvironment-derived lactate activated PGC1α-mediated oxidation [48]. PPARγ coactivator 1α (PGC1α) is a multifunctional transcriptional regulator that induces mitochondrial metabolism enzymes, which potentiate CRC malignant progression and support chemotherapy resistance [49]. Moreover, lactate affects cell fate by participating in a novel protein modification, “lactylation” [50].

### TCA cycle

The TCA cycle is a metabolic hub in mitochondria that can produce various metabolic intermediates and NADH to support cell growth. Pyruvate produced through glycolysis is catalyzed into acetyl-CoA via the PDH (pyruvate dehydrogenase) complex and then enters the TCA cycle. Except for the steps catalyzed by citrate synthase and the OGDH (oxoglutarate dehydrogenase) complex, most of the reactions are reversible, and the bulk of the intermediates can be replenished by anaplerotic reactions [51] (Fig. 1). TCA cycle flux plays a vital role in cellular metabolism and is frequently dysregulated in cancers, including CRC [49, 52].

PDH regulates the conversion of pyruvate to acetyl-CoA, but is inhibited when phosphorylated by PDK1 (pyruvate dehydrogenase kinase 1). PGK1 (phosphoglycerate kinase 1), the first glycolytic ATP-producing enzyme, prevents glucose flux into the TCA cycle and, in contrast, generates increasing levels of lactate. Studies have illustrated that O-GlcNAcylation of PGK1 at T255 activates its kinase activity and induces PGK1 translocation into mitochondria, thereby coordinating glycolysis and the TCA cycle to promote tumor development via PGK1-mediated PDH phosphorylation [53]. The suppressor wild-type P53 transcriptionally inhibits PDK2 to ameliorate tumorigenesis, but in cancer cells with P53 deficiency, PDK2 is dysregulated, and glucose is redirected for lactate production [54]. Meanwhile, the small-molecule DCA (dichloroacetate) directly activates wild-type P53 to attenuate CRC growth and restores the 5-FU sensitivity of CRC. DCA treatment upregulates wide-type P53 and then upregulates mir-149-3p to target the 3'-UTR of PDK2 to inhibit its expression [55]. Wnt/β-catenin signaling directly increases PDK1 transcription to block TCA flux and facilitate the Warburg effect [56].

IDH (isocitrate dehydrogenase) converts isocitrate to α-KG (α-ketoglutarate), a tumor-suppressing metabolite of P53, which compensates for P53 deficiency to arrest malignant progression [57]. IDH1 is frequently hyperacetylated in CRC. SIRTs (sirtuins) constitute a class of deacetylases with various biological activities. SIRT2 is a deacetylase of IDH1, and hyperactivated SIRT2 blocks the progression of CRC through SIRT2-dependent IDH1 K224 deacetylation [58]. Under hypoxic conditions, IDH1 promotes the formation of glutamine-derived acetyl-CoA through a reduction pathway and redirects carbon flux to lipogenesis to compensate for the loss of intracellular macromolecules. Reductive TCA metabolism abrogates PDH enzyme activity via HIF/PDK1/PDH signaling and subsequently decreases citrate production, while IDH1 generates citrate from glutamine-derived α-KG via reduction to maintain lipogenesis [10]. The abundance of IDH1 protein has been negatively correlated with Skp2. Mechanistically, Skp2 destabilizes IDH1 to mediate cell cycle progression, resulting in suppression of the TCA cycle in the S phase [59]. Additionally, elevated IDH2 has been observed in CRC, in which it activates the TCA cycle to support NADPH production and thus prevent ROS-induced DNA damage [60]. However, functional mutations in IDH1/2 enhance the accumulation of R-2HG (R-2-hydroxyglutarate), which suppresses the activation of α-KG-dependent enzymes [61]. IDH1^R132H^ mutant cells accumulate R-2HG, which can directly bind and inhibit the ATP synthase β subunit, leading to mitochondrial disorder and inhibiting mTORC1 signaling [62].

The enzyme OGDH oxidizes the conversion of α-KG to succinate and is located mainly in the nucleus and mitochondria. Nuclear OGDH couples with the histone acetyltransferase KAT2A to form the OGDH-KAT2A complex, which serves as a histone H3 succinyltransferase that mediates histone K79 modification and local generation of succinyl-CoA to promote CRC [63]. Oncogenic PIK3CA mutations are commonly found in cancers and render CRC cells increasingly dependent on OGDH-related glucose metabolism, which indicates a precise metabolic vulnerability to synthetic lethality [64]. Under hypoxic conditions, activated HIF-1 facilitates E3 ligase SIAH2-targeted ubiquitination and proteolysis of the E1 subunit of the OGDH complex, resulting in glucose flux diversion from the TCA cycle to lipogenesis [65].

ME (malic enzyme) converts malic acid into pyruvate, and together with G6PD, it is the main enzyme that provides NADPH to eliminate ROS. Upregulated ME1 in KRAS^G13D^ mutant cells is indispensable for cancer development, while miR-30a directly inhibits ME1 expression [66]. During P53-mediated senescence progression, P53 downregulates both ME1 and ME2 to maintain cellular senescence-type metabolism, and downregulation of ME1 and ME2 is mutually activated via P53 through a feed-forward mechanism mediated by MDM2 and AMPK [67]. However, in P53-mutant cancer cells, ME2 enhances the production of the “oncometabolite” 2-HG and prevents MDM2-mediated P53 degradation by binding to mutant P53, facilitating P53 mutant-driven tumor growth [68]. Additionally, β-catenin transcriptionally increases PGAM5 and ME1 levels, and elevated phosphatase PGAM5 mediates ME1 S336 dephosphorylation to trigger ACAT1-mediated ME1 K337 acetylation; both of these events activate ME1. However, ME1 S336 dephosphorylation and K337 acetylation can be reversed by NEK1 kinase and SIRT6 deacetylase, respectively [69].

### The pentose phosphate pathway

The PPP is a branch pathway of glycolysis and is the main pathway through which intracellular NADPH is produced, and NADPH thus produced reacts in the cytoplasm and is activated in various cancers, including CRC. Through oxidative and nonoxidative steps, glycolytic G6P is converted to ribose-5-phosphate for the de novo synthesis of nucleotides (Fig. 1). Moreover, NADPH from the PPP is indispensable for the generation of reduced GSH (glutathione), a major ROS scavenger [16]. Accumulation of intracellular ROS interferes with the biomass levels of lipids, proteins, and nucleotides, while NADPH provides the reducing power for the GSH system to eliminate ROS [70]. Hence, the PPP is essential for supporting CRC cell growth and producing NADPH to overcome oxidative stress.

G6PD (6-Phosphogluconate dehydrogenase), PGLS (6-phosphogluconolactonase), and 6PGD (6-phosphogluconate dehydrogenase) catalyze the oxidative PPP branch, which is closely linked to tumor growth. High G6PD expression in CRC maintains redox homeostasis, and altered G6PD is partially stimulated via c-Src-mediated G6PD Y112 phosphorylation at the posttranscriptional level and YY1- or NeuroD1-mediated G6PD upregulation at the transcriptional level [71–73]. Moreover, P53 family proteins are involved in G6PD regulation. Formation of the P53-G6PD complex prevents the formation of the active G6PD dimer via a competition mechanism, suppressing PPP flux to inhibit NADPH production [8]. In contrast, TAp73 confers a proliferative advantage to CRC cells, as it transcriptionally activates G6PD and drives PPP flux to generate NADPH and ribose that is consumed in the biosynthesis of macromolecules and ROS detoxification [74]. However, G6PD-mediated carcinogenic effects are dispensable in *KRAS*-mutant cancer cells [75]. Interestingly, 6PGD forms a complex with ME1 that enhances the binding affinity of 6PGD for substrates, thereby enhancing PPP flux and leading to NADPH accumulation and tumor development [76]. 6PGD-driven production of ribulose-5-phosphate inhibits AMPK activation by disrupting LKB1 (liver kinase B1) complex activation, thereby activating lipogenesis and increasing tumor growth [77]. PGLS deletion increases the 6-phosphogluconolactone level but decreases the ribulose-5-phosphate level, which enhances the c-Src-mediated inhibitory Y307 phosphorylation of PP2A (protein phosphatase 2A) to increase AMPK phosphorylation [78]. Together, PGLS and 6PGD regulate AMPK homeostasis by balancing the opposing LKB1 and PP2A signaling [77, 78].

TKT (transketolase) and RPIA (ribose-5-phosphate isomerase A) are enzymes in the nonoxidative PPP branch. TKT is linked to poor prognosis for CRC patients and participates in glycolysis-mediated metastasis by activating GPR78-induced AKT phosphorylation [79]. Additionally, SIRT5 activates TKT to maintain the nucleotide pool by increasing TKT L281 demalonylation [80]. In CRC, the noncanonical metabolic protein RPIA forms a hetero-oligomer with β-catenin to maintain β-catenin activity and thus promote tumorigenesis [81]. However, miRNA-124 targets RPIA mRNA, which blocks PPP flux to attenuate cell proliferation [82].

## Amino acid metabolism in CRC

**Fig. 2:**
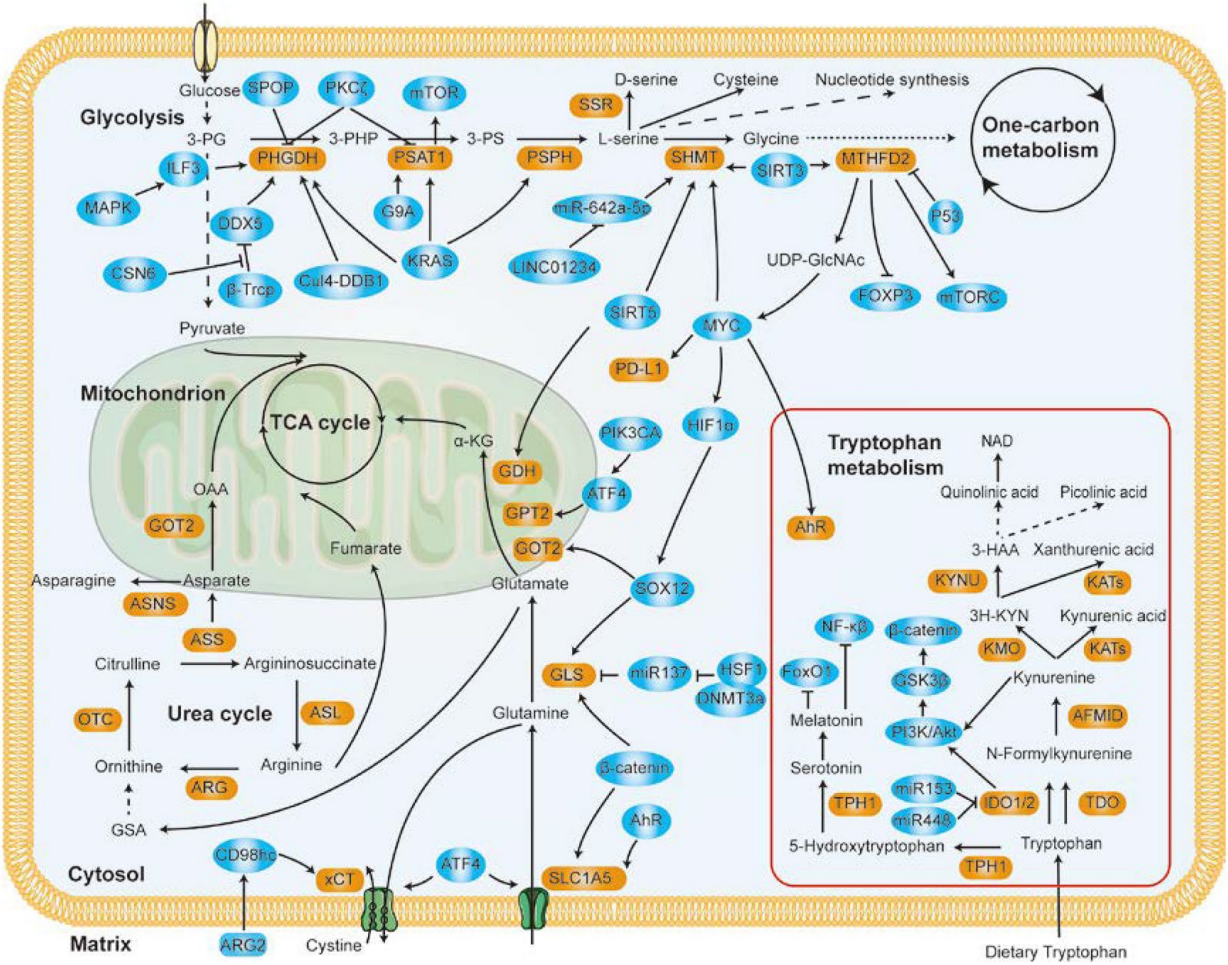
Amino Acid Metabolism in Cancer Cells and Its Crosstalk with Other Signal Pathways. Exogenous and endogenous amino acids are metabolized in CRC cells to fuel the unique biosynthetic and energetic requirements of the tumor, and metabolic enzymes involved in amino acid metabolism are regulated y different signals. Brown boxes indicate metabolic enzymes. Blue boxes represent factors that alter amino acid metabolism

Amino acids are the building blocks of proteins and are key factors in multiple anabolic processes that fuel cancer cell growth [83]. Metabolism tracing has shown that exogenous amino acids are the most important factors contributing to the mass of proliferating tumor cells [84]. Amino acids have been classified into nonessential amino acids and essential amino acids, and research on glutamine, serine, and glycine has been a focus. Dietary serine, glycine, and methionine starvation shrunk tumor growth in vivo, which shows that amino acids are indispensable for tumor growth [85, 86]. Moreover, genetic mutation regulates the expression and function of amino acid enzymes, satisfying the metabolic demands for cancer cell growth [85]. Hence, the specific relationship between amino acid metabolism and malignant tumor progression is summarized in Fig. 2.

### Glutamate and glutamine metabolism

In a compensatory mechanism to counteract deficient mitochondrial function, glutamine metabolism is frequently dysregulated in cancer cells [87]. Normally, the transporters SLC1A5 and SLC7A5 mediate the absorption and efflux of glutamine. Glutamine is transformed into glutamate by GLS (glutaminase) and then converted to α-KG via GPT (glutamate pyruvate transaminase), GOT (glutamate oxaloacetate transaminase), or GDH (glutamate dehydrogenase) [88] (Fig. 2). Glutamine metabolism contributes to the continuous growth capacity of cancer cells by fueling the production of ATP and biosynthesis of bioactive molecules by replenishing TCA cycle metabolites. However, the depletion of glutamine disrupts cancer development owing to the glutamine addiction of these cells [89, 90]. Glutamine starvation can induce a GCN2 (general control non-depressible 2)-mediated integrated stress response that enables cells to adapt to metabolic stress. Activation of the signal integrator GCN2 facilitates tumor cells escape from MYC-driven apoptosis via an MYC/GCN2/eIF2α negative feedback loop [91].

Oncogenic GLS1 hydrolyzes glutamine into glutamate and fuels CRC cell growth [92]. During CRC development, HIF-1α binds to the SOX12 (SRY-box transcription factor 12) promoter to increase SOX12 expression, which further increases the SOX12-mediated expression of GLS and GOT2, leading to cellular metabolism adaptation that promotes tumor progression [93]. HSF1 (heat shock factor 1) exhibits protumor properties in a GLS1-dependent manner. Elevated HSF1 recruits DNMT3a to suppress the expression of miR137, which targets GLS1 mRNA, stimulating GLS1-dependent mTOR activation to promote CRC development [94]. The amino acid sensor ATF4 is also involved in the regulation of amino acid metabolism. Stress-induced activation of ATF4 directly increases the expression of SLC1A5 and GPT2 by binding to their promoters [95, 96]. Moreover, genetic PIK3CA^p110α^ contributes to reprogrammed glutamine metabolism via the p110α/ATF4/GPT2 axis, which renders CRC cells more addicted to glutamine [89]. Elevated GPT2 promotes α-KG production from glutamate, accelerating glutamine carbon entry into the TCA cycle to promote tumorigenesis [90]. Mitochondrial SIRT5 supports the anaplerotic entry of glutamine into the TCA cycle in CRC via its activation of GDH1 in a deglutarylation-dependent manner [97].

Glutamine transporters mediate intracellular glutamine homeostasis and establish a complex regulatory network. SLC1A5 and SLC7A5 are important to cell growth, and the transcription factor AhR (aryl hydrocarbon receptor) increases their expression by binding to their respective promoters [98]. The KRAS^G12D^ mutation contributes to glutamine metabolism, leading to the activation of oncogenic signaling but a reduction in ROS levels, while the deletion of SLC7A5 or SLC25A22 attenuates KRAS^G12D^-driven tumorigenesis [99, 100]. During CEMIP (cell migration-inducing and hyaluronan-binding protein)-mediated CRC metastasis, CEMIP promotes the translocation of β-catenin and reprograms glutamine metabolism by increasing GLS1 and SLC1A5 protein expression [101]. Furthermore, the cystine/glutamate antiporter xCT enhances the production of the antioxidant GSH to support KRAS^V12^-induced tumorigenicity via the KRAS^V12^-induced ETS1-ATF4 collaborative system-mediated upregulation of xCT [102]. Interestingly, the membrane protein CD98hc activated by ARG2 secreted from tumor-associated neutrophils increases the rate of xCT transport to promote CRC liver metastasis[103].

### Serine-glycine-one-carbon metabolism

Serine and glycine are nonessential amino acids and the main sources of one-carbon donors, which are required for GSH biosynthesis and other cellular metabolism processes [104]. Physiological levels of serine and glycine are insufficient to promote tumorigenesis and malignancy, but biosynthesis pathways that are frequently activated in cancer cells in advantageous to tumor growth and drug resistance [105–107]. The SSP (serine synthesis pathway), glycine synthesis pathway, and the one-carbon cycle called the SGOC (serine-glycine-one-carbon) pathway, contributes to a complex metabolic network (Fig. 2). SGOC pathway activation leading to the uptake, biosynthesis, and conversion of serine and glycine is frequently amplified in cancer cells and is therefore essential to cancer biology [104].

PHGDH (phosphoglycerate dehydrogenase) and PSAT (phosphoserine aminotransferase) are required for serine metabolism. PHGDH diverts the glycolytic 3-phosphoglycerate into the SSP and the expression of which is amplified in CRC [108]. However, the glycolytic enzyme PKM2 switches the direction of metabolic flux, driving increased SSP activation by controlling the availability of 3-phosphoglycerate [109]. ILF3 (interleukin enhancer-binding factor 3) is a double-stranded RNA-binding protein that stabilizes SSP-related gene mRNA to stimulate serine biosynthesis, and ILF3-mediated SSP activation can be promoted by MAPK phosphorylation of ILF3, while E3 ligase SPOP-dependent degradation of ILF3 breaks SSP activation [108]. Similarly, the Cul4A-DDB1 E3 ligase complex induces PHGDH K146 monoubiquitination, enhancing PHGDH enzyme activity from mutant low-activity PHGDH K146R, which promotes CRC metastasis progression by increasing intracellular S-adenosylmethionine levels and initiating SETD1A-mediated histone methylation of LAMC2 and CYR61[110]. Furthermore, CSN6 prevents E3 ligase β-Trcp-mediated DDX5 proteasome degradation, which in turn stables PHGDH mRNA in a DDX5-dependent manner [111]. Elevated PSAT1 implicates cell metabolism contributions to CRC cell growth and chemoresistance [106, 112]. Histone methyltransferase G9A increases PSAT1 expression by increasing PSAT1 H3K9me1 levels, which activates the SSP and provides α-KG that enters the TCA cycle, thereby sustaining cancer cell proliferation [113]. Silencing of PSAT1, in turn, induces the degradation of cyclin D1 via PSAT1-mediated phosphorylation of mTOR and S6K, which gives rise to cell cycle arrest and necrocytosis [113, 114].

Cancer cells also show metabolic plasticity through which they adapt to nutrient stress and maintain malignant properties. Genetic KRAS^G12D^ mutation increases the expression of SSP enzymes to promote de novo serine synthesis [85]. Moreover, serine starvation can stimulate the stress protein ATF4 to activate the SSP by increasing the expression of SSP-related genes [114, 115]. Furthermore, serine starvation-mediated SSP activation induces P53-dependent metabolic remodeling to meet the anabolic demands of tumor cell proliferation [116]. PKCζ, a member of the PKC family, targets proteins related to cellular metabolism to suppress CRC. For example, PKCζ blocks SSP activation by suppressing PHGDH and PSAT1 activity, and PKCζ phosphorylates PHGDH at T57/T78 to inhibit its enzymatic activity, while PKCζ deletion restores the necessary metabolic processes mediated through the SSP in starved cancer cells [117]. Serine starvation also activates serine palmitoyltransferase to induce the accumulation of 1-deoxysphingolipids, alanine-derived toxic sphingolipids, resulting in inhibited tumor growth [118]. The fine-tuning of serine trafficking and consumption within cancer cells enables the integration of different types of signals, which may be the reason that serine starvation determines cell fate to survival or death [116, 118].

Glycine is related to the rapid proliferation of CRC cells [107]. In mitochondria, SHMT (serine hydroxymethyltransferase) initiates the degradation of serine and results in the production of glycine and NADPH. Two SHMT isozymes have been identified in mammals, SHMT1 (cytoplasmic isozyme) and SHMT2 (mitochondrial one). Both SHMT1 and SHMT2 play important roles in CRC. Deletion of SHMT2 induces respiratory chain dysfunction and suppresses cancer cell growth, while double knockout of SHMT1/2 almost completely inhibits tumorigenesis [119, 120]. Under hypoxic stress, activated HIF1α enhances the expression of SHMT2 in an MYC-dependent manner, which supports tumor growth by producing NADPH to counterbalance redox stress [121]. SIRT3- and SIRT5-mediated deacetylation is involved in SHMT activity. SIRT5 desuccinylates SHMT2 at K280, and SIRT3 deacetylates SHMT2 at K95, and both of these modifications activate SHMT2 enzyme activity, subsequently driving the degradation of serine to induce CRC carcinogenesis [122, 123]. In addition, the lncRNA LINC01234 modulates CRC progression in an SHMT2-dependent manner. Overexpressed LINC01234 in CRC cells competes with miR-642a-5p for the binding sites on SHMT2, which results in the upregulation of SHMT2, thereby promoting CRC cell growth [124].

One-carbon metabolism integrates signals involved in cellular biological progress, including signals involved in cellular metabolism, methylation, and ROS reduction [104]. Serine and glycine contribute to one-carbon metabolism via SHMT-mediated production of methylenetetrahydrofolate, which is further catalyzed by MTHFD (methylene-tetrahydrofolate dehydrogenase). During CRC progression, oncogenic *KRAS* activates AKT and ERK signaling, increasing c-Myc-mediated MTHFD2 expression to maintain NADPH homeostasis, which has been linked to cell survival [125]. Similarly, in cancer cells with P53 deficiency, MTHFD2 binds PARP3 to prevent DNA damage [126]. In CRC cells, SIRT3 downregulates K88 acetylation of MTHFD2 to maintain MTHFD2 enzyme activity and NADPH production, and cisplatin disrupts these processes by inhibiting SIRT3 expression [127]. In addition to generating NADPH to reduce ROS levels, SGOC metabolism produces NADH in respiration-insufficient cancer cells. Accumulation of NADH is toxic to cells with impaired respiration, which can impede tumor progression [128]. Additionally, MTHFD2 is a metabolic checkpoint in CRC immunotherapy. MTHFD2 drives de novo purine synthesis to maintain CD4^+^ T cell proliferation and inflammatory cytokine production, contributing to regulatory T-cell fate and function [129]. Moreover, MTHFD2 sustains cellular UDP-GlcNAc levels to increase c-Myc O-GlcNAcylation, which enhances PD-L1 transcription, contributing to tumor resistance against T-cell cytotoxicity [130]. MTHFD2 deficiency, however, impedes Th17 cell function by increasing FOXP3 expression and dampening mTORC1 activity [129].

### Tryptophan metabolism

The essential amino acid tryptophan is the only amino acid with an indole structure. Three distinct pathways are responsible for tryptophan metabolism and catabolize tryptophan to produce bioactive molecules, such as serotonin, melatonin, nicotinamide, and VB_3_ [131] (Fig. 2). Meanwhile, intermediates of tryptophan show distinct content changes and functions in carcinogenesis [131]. Undoubtedly, tryptophan is essential for cell survival and is actively taken up and converted to other bioactive molecules in CRC cells [132, 133].

The AhR pathway, which is important for sensing kynurenine metabolites to regulate CRC pathological progression, is processed by the gut microbiota [134]. AhR was initially found to mediate the toxic effects of 2,3,7,8-tetrachlorodibenzo-p-dioxin and to be overexpressed in human CRC cells [135]. The ligands of AhR, which are derived from microbial tryptophan catabolites, play distinct roles in health and diseases [136]. For instance, an in vivo study showed that the activation of the AhR pathway mediated via tryptophan-derived metabolites contributed to a high risk of venous thrombogenicity [137]. Furthermore, MYC-driven tryptophan catabolism activates the production of kynurenine, promoting the nuclear translocation of AhR to promote tumor growth [132].

The serotonin pathway generates serotonin in enterochromaffin cells via the action of TPH1 (tryptophan hydroxylase 1). TPH1-mediated serotonin generation protects against colorectal carcinogenesis by promoting tissue-specific DNA repair activity in colonic crypts [138]. Melatonin, a downstream serotonin product, exerts antitumor effects. Mechanistically, melatonin treatment inhibits both FoxO1 and NF-κβ activity, which positively regulates endothelin-1 promoter activity, thereby blocking CRC progression via melatonin-reduced endothelin-1 expression [139]. Taken together, studies have shown that the serotonin pathway may be a useful targetable metabolic venerability for cancer.

The kynurenine pathway is initiated by IDO1 (indoleamine 2,3-dioxygenase 1). Elevated IDO1 levels are commonly observed in CRC and have been highly correlated with poor prognosis [140, 141]. Moreover, increased IDO1 is thought to reduce the availability of tryptophan in the tumor microenvironment, thereby decreasing the immune response and ultimately promoting tumorigenesis [141–143]. Surprisingly, the regulatory effects of miRNAs on IDO1 have been identified in CRC and immune cells; that is, miR153 and miR448 targeted the 3'-UTR of IDO1 to suppress IDO1 expression and potentiate immune cytotoxic functions [142, 143]. IDO1 is undoubtedly a stimulator of CRC progression, and IDO1 downstream metabolites regulate tumor cell growth. Collectively, the data suggest that kynurenine pathway metabolites disrupt the stability of GSK-3β and activate PI3K/AKT signaling in CRC cells, which together contribute to the nuclear translocation of β-catenin and subsequently drive cell proliferation [140, 144].

## The role played by lipid metabolism in CRC

**Fig. 3:**
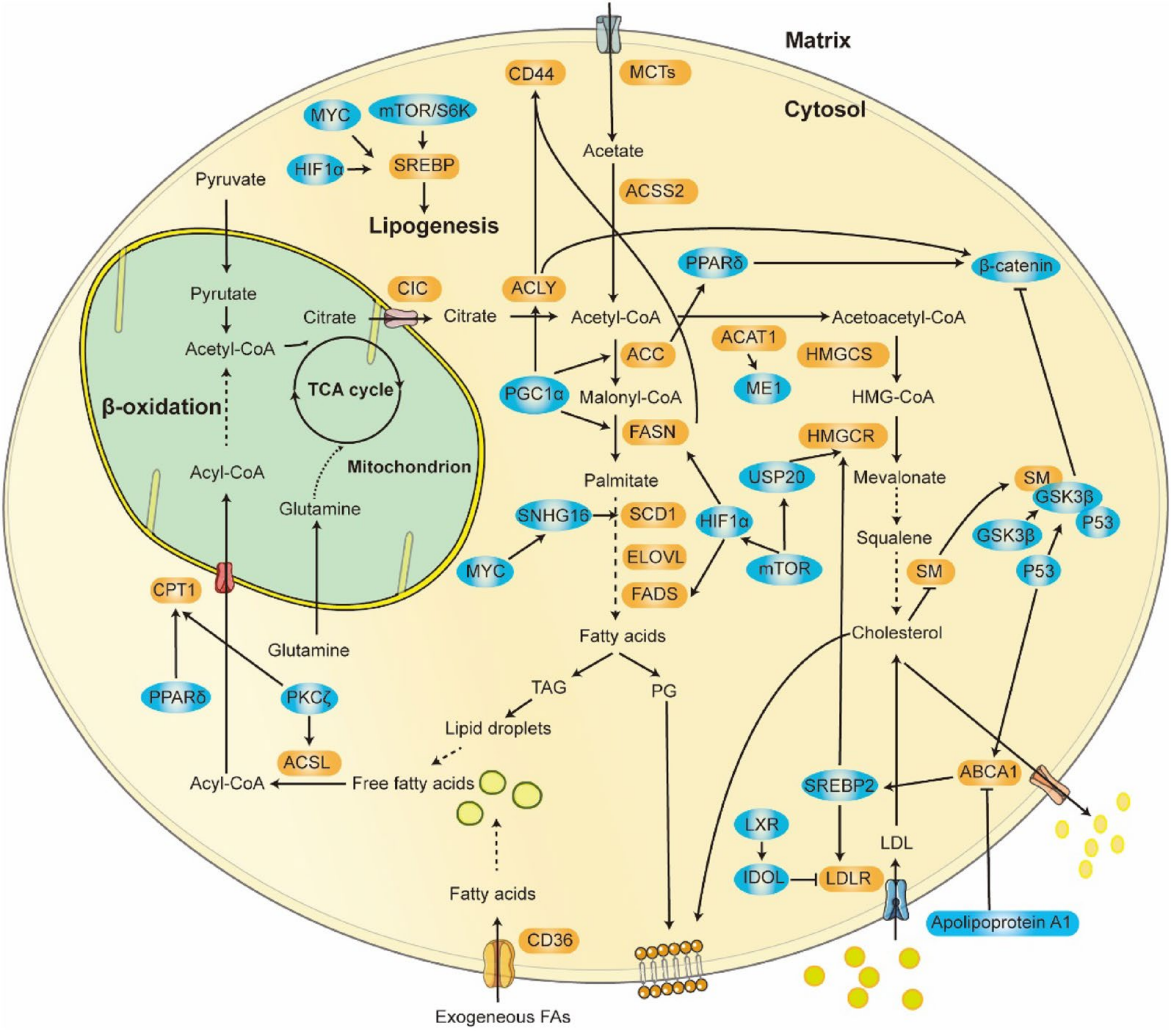
Dysregulation of Lipid Metabolism Pathways in CRC Cells and Their Metabolic Enzymes with Regulatory Networks. For de novo lipogenesis, CRC cells rely on glucose, glutamine, and acetate to synthesize acetyl-CoA. This is further catabolized by multiple enzymatic reactions to synthesize fatty acids and cholesterol, which provide a range of fatty acids to meet the cell requirements. In turn, excess fatty acids are oxidated to provide energy under stress conditions. Brown boxes indicate metabolic enzymes. Blue boxes represent factors that alter lipid metabolism

Numerous lipids are present in our bodies, and they perform various physiological and pathological functions. Lipids are mostly composed of fatty acids, which are formed by a carboxylic acid group and hydrocarbon chains of various lengths with different degrees of desaturation [145]. Even though lipids are found primarily in adipose tissue, they also function abnormally to mediate cancer cell development. In cancer cells, lipids are typically stored in LDs (lipid droplets), and the accumulation of LDs contributes to CRC pathological function [146]. Cholesterol functions as a membrane component or signaling factor that regulates cell processes linked to CRC development [147]. Aberrantly altered lipogenic enzymes increase the function of lipogenesis and enhance the uptake of exogenous lipids, which is characteristic of cancer cells. Here, we focus on aberrant regulation of fatty acid and cholesterol metabolism in CRC, which helps to better understand tumor metabolism (Fig. 3).

### Fatty acid metabolism

Deregulated fatty acid metabolism is evident in many tumors. De novo lipogenesis and FAO (fatty acid oxidation) are two mechanisms that maintain cellular lipid homeostasis in the cytoplasm and mitochondria, respectively [148]. Multiple lipogenic enzymes control the synthesis, elongation, and desaturation during de novo lipid synthesis, simultaneously contributing to cellular processes linked to malignancy progression [148]. FAO mediates the degradation of fatty acids and generates energy for promoting cancer cell growth, survival, and metastasis [149, 150] (Fig. 3).

ACLY (ATP citrate lyase) mediates the first step of de novo lipogenesis, converting citrate into acetyl-CoA and oxaloacetate. The transmembrane protein CD44 is connected to tumor metastasis. A study demonstrated that blocking ACLY activity significantly lowered the expression of CD44 and downstream signaling, which contributed to the prevention of CRC development [151]. In addition, through direct interactions, ACLY facilitates the nuclear import of β-catenin subsequently enhancing the β-catenin transcriptional activity and CRC metastasis [152]. Besides the regulation of OXPHOS, PGC1α promotes lipogenesis by inducing the expression of lipogenic genes (ACLY, ACC, and FASN), thereby increasing fatty acid synthesis to promote tumor growth [153].

ACC (acetyl-CoA carboxylase) converts acetyl-CoA to malonyl-CoA and is associated with a poor prognosis in CRC. PPARδ (peroxisome proliferator-activated receptor δ) is a nuclear receptor that senses cellular lipid levels to regulate lipogenesis. In intestinal epithelial cells, functional ACC1 drives de novo lipogenesis to promote the nuclear localization of PPARδ, which interacts with β-catenin to maintain Wnt-mediated stem cell function and is thus involved in the progression of CRC [154].

FASN (fatty acid synthase) is responsible for the production of palmitate, and the expression of FASN progressively increases in advancing stages of CRC [151]. In addition to ACLY/CD44 signaling, FASN is involved in CD44-mediated metastasis. An enhanced FASN level increases the expression of CD44 and subsequently activates CD44-associated signaling, contributing to the promotion of the migration and adhesion of CRC cells [151]. Tumor-associated fibroblasts also potentiate CRC development. FASN-mediated metabolic reprogramming in fibroblasts increases the secretion of fatty acids and phospholipids, which are absorbed by CRC cells to fuel their malignant progression [155].

ACSL (acyl-CoA synthetase) and SCD (stearoyl-CoA desaturase) lipogenic metabolic networks are widely regulated in CRC. ACSL is critical for the conversion of FAs to FA-CoA, and SCD controls the saturation and desaturation of fatty acids. When treated with palmitate, the kinase PKCζ phosphorylates SIRT6 at T294, which in turn induces the transcription of ACSL1 and CPT1, thereby activating FAO and indicating a novel role for PKCζ in lipid homeostasis [156]. In contrast to ACSL1, SCD1 is a putative protumor or antitumor factor in CRC, and cancer cells show sufficient metabolic plasticity to adapt to the SCD1 status and maintain their proliferative capacity. Upregulated SCD1 promotes CRC metastatic progression by increasing the production of oleic acid [157]. In contrast, intestinal SCD1 deficiency favored CRC progression in mice, while oleic acid reduced the tumor burden [158]. Deletion of SCD1 in the intestine and the subsequent absence of oleic acid induce the establishment of a proinflammatory environment, which can promote cell proliferation and cancer development mediated via the paracrine action of cytokines [158]. In CRC cells, MYC-induced lncRNA SNHG16 is a competing endogenous RNA sponging miRNA that targets the 3'-UTR of SCD1, which increases the activity of SCD1 [159].

CPT1 (carnitine palmitoyltransferase 1) transports fatty acids from the cytosol to mitochondria for consumption during FAO. Three CPT1 subtypes have been identified in mammals, CPT1A, CPT1B, and CPT1C (brain). CPT1A is upregulated in advanced metastatic tumors, suggesting that CPT1A may be a useful target for metastatic CRC treatment [150]. Upregulated CPT1A in detached CRC cells contributes to FAO and subsequently induces anoikis resistance, which in turn enhances the metastatic capacity of CRC cells [150]. Adipocytes from adipose tissues adjacent to CRC tissues fueled tumor growth in PPARδ-mediated FAO. Exogenous adipocytes or fatty acids activate PPARδ, increasing CPT1A at the transcriptional level and stimulating Wnt/β-catenin signaling to promote CRC progression [160]. Consistent with a fibroblast-CRC model, an adipocyte-CRC model showed progressive protumor effects mediated via its secreted metabolites, which confirmed the important role played by lipid metabolism in CRC [155, 160]. Cancer cells often favor de novo lipogenesis while suppressing FAO to maintain their energetic demands under hypoxic conditions. Activated HIF1α transcriptionally regulates cellular metabolism by upregulating FASN and SREBP1 expression but downregulating CPT1A, resulting in enhanced lipid uptake and transport [161].

SREBPs (sterol regulatory element-binding proteins) regulate the expression of genes involved in lipid metabolism. SREBP1a, SREBP1c, and SREBP2 are three SREBP isoform proteins that have come to light in humans. They play different roles in lipogenesis: SREBP1a initiates the biosynthesis of fatty acids and cholesterol, SREBP1c is involved in fatty acid synthesis, and SREBP2 regulates cholesterol metabolism [162]. SREBPs control lipogenesis at both the transcriptional and posttranscriptional levels. Notably, mTORC1 integrates cellular anabolic signals to promote lipogenesis. The expression of lipogenic enzyme transcripts is increased by mTORC1-induced SREBP activation [163]. Moreover, mTORC1-activated S6K1 phosphorylates SRPK2 at S494 and promotes CK1 phosphorylation of SRPK2 at S497. Then, phosphorylated-SRPK2 is translocated into the nucleus to promote SR protein (a splicing regulator for lipogenic genes) binding to U1-70 k and induce the splicing of mTORC1/SREBP-induced lipogenic pre-mRNAs, which in turn promotes the maturation of lipogenic proteins via mTORC-mediated posttranscriptional modification [163]. Furthermore, MYC increases SREBP1 transcription, and MYC cooperates with SREBP1 at these MYC-elevated levels to induce the transcription of lipogenic genes, thereby promoting MYC-induced tumorigenesis and tumor progression [164].

### Cholesterol metabolism

Cholesterol plays an important physiological function in the human body, as a component of the cell membrane, and lipoproteins. Tumorigenesis drives the synthesis of endogenous cholesterol and metabolizes it to fuel rapid cell proliferation [165]. Cholesterol metabolism and CRC are intricately linked, and elevated total cholesterol levels are associated with an increased risk of CRC development [166] (Fig. 3).

ACAT1 (acetyl-CoA acetyltransferase 1) is responsible for the transformation of cholesterol into cholesteryl esters and regulates the production of cellular cholesterol. The ACAT1 holoenzyme is a tetramer that comprises two homodimers, with each dimer acting as a catalytic unit [167, 168]. Furthermore, ACAT1 acetylates ME1, cooperates with ME1 to form a dimer, and thus activates ME1 to regulate the homeostasis of NADPH and cholesterol metabolism [69].

HMGCR (hydroxymethylglutaryl-CoA reductase) is the rate-limiting enzyme involved in the mevalonate pathway. SREBP2 regulates the transcription of HMGCR and LDLR (low-density lipoprotein receptor) to maintain cholesterol homeostasis [169]. Squalene monooxygenase, another rate-limiting enzyme that, beyond HMGCR, is associated with metastatic CRC. However, accumulated intracellular cholesterol induces squalene monooxygenase degradation and promotes CRC progression by activating Wnt signaling and deactivating P53 signaling [170].

LXR (liver X receptor) is a transcriptional regulator of cholesterol metabolism and inhibits CRC progression [171]. In addition to the promotion of cholesterol efflux, LXR regulates the uptake of exogenous cholesterol. Mechanistically, LXR triggers IDOL-mediated ubiquitination-related degradation of LDLR. LXR increases the expression of IDOL, an E3 ubiquitin ligase of LDLR, which induces the degradation of LDLR, thereby limiting the uptake of LDL to maintain cholesterol homeostasis [172]. In cooperation, LXR signaling and SREBP signaling control sterol level-based regulation of cholesterol uptake [162, 172]. Pyroptosis is an inflammation-related type of cell death, and LXRβ-induced pyroptosis has been observed in cancer cells. Activated LXRβ triggers the pannexin 1 channel to release ATP and specifically induces caspase-1-dependent CRC cell death via pyroptosis [173]. Additionally, energy stress caused by LXRβ activation results in an integrated stress response, which leads to the inhibition of BCL-2 family proteins and cancer cell apoptosis [174].

ABCA1 (ATP-binding cassette transporter A1) mediates the efflux of cholesterol and regulates cellular cholesterol homeostasis. In CRC cells, ABCA1 creates a bond between P53 and SREBP2. Activated P53 induces ABCA1 transcription, which inactivates SREBP2 and inhibits tumor growth [175]. Apolipoprotein A1 is the main protein component of plasma high-density lipoprotein. Overexpressed apolipoprotein A1 has been reported to attenuate the acquisition of malignant phenotypes driven by ABCA1 to compensate for excessive ABCA1-dependent efflux of cholesterol in CRC [176].

Diet is also involved in the regulation of cholesterol metabolism. High-volume feeding has been demonstrated to trigger cholesterol biosynthesis via the mTORC1/USP20 (ubiquitin-specific peptidase 20) /HMGCR axis. In cancer cells, feeding-induced nutrient stress caused the accumulation of insulin and glucose, which stimulated mTORC1 to phosphorylate USP20. HMGCR recruited phosphorylated USP20 to form the HMGCR-USP20 complex, which protected HMGCR from proteasome-mediated degradation [177]. While feeding caused tumors to grow, fasting exerted an antitumorigenic effect by targeting FDFT1 (farnesyl-diphosphate farnesyltransferase 1)-mediated condensation of farnesyl pyrophosphate. Mechanistically, fasting upregulated the level of FDFT1, which in turn resulted in the suppression of the AKT/mTOR/HIF1α pathway, thereby inhibiting CRC cell growth in response to FDFT1-inhibited glycolysiss [178].

## Effects of nucleotide metabolism in CRC

**Fig. 4:**
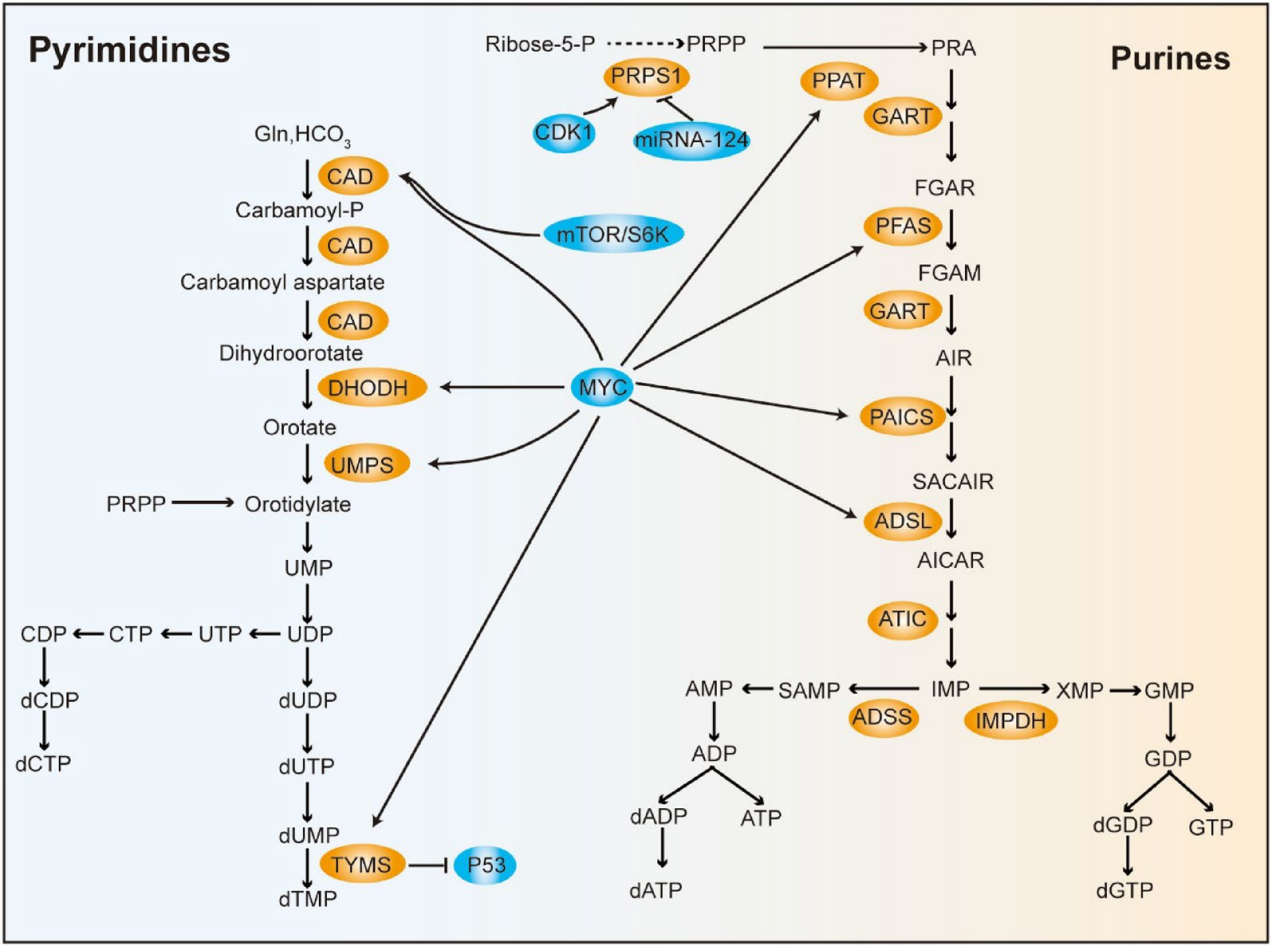
Simplified Schematic of the De Novo Pyrimidine and Purine Synthesis Pathways in CRC Cells. Phosphoribose-1-pyrophosphate (PRPP) derived from the PPP pathway participates in the synthesis of pyrimidine and purine via distinct pathways with different substrates. Brown boxes indicate metabolic enzymes. Blue boxes represent factors that alter nucleotide metabolism

Nucleotides are involved in a wide range of biological processes and are constantly synthesized through endogenous mechanisms in cancer cells. Nucleotide biosynthesis is aligned with cancer-related metabolic alterations such as augmented aerobic glycolysis and increased lipogenesis in CRC [179, 180]. Normally, cellular purine and pyrimidine pools are maintained through de novo synthesis and salvage pathways, and proliferating cells, such as cancer cells tend to show upregulated activation of de novo synthesis pathways [181]. Through reactions catalyzed by many different metabolic enzymes, nucleotides are ultimately generated to meet the needs of cancer cells (Fig. 4).

PRPS1 (ribose-phosphate diphosphokinase 1) converts ribose-5-phosphate into 5-phosphoribosyl-1-pyrophosphate. CDK1 phosphorylates PRPS1 at S103 to fuel nucleotide synthesis and induce cell cycle progression, while miRNA-124 targets PRPS1 expression [82, 182]. CAD (carbamoyl phosphate synthetase/aspartate transcarbamylase/dihydroorotase)-dependent nucleotide metabolic flux is important to CRC. Aspartate availability for CAD is competitively regulated via the urea cycle. Silencing of the urea cycle enzyme argininosuccinate synthase 1 leads to aspartate consumption for pyrimidine synthesis and stimulates mTOR-phosphorylated S6K to activate CAD [183]. DHODH (dihydroorotate dehydrogenase) produces orotate and is linked to CRC [179]. As a component of the mitochondrial electron transport chain, DHODH is necessary for tumor growth [184]. DHODH is the key mediator responsible for inhibiting cancer cell mitochondrial ferroptosis [185]. Methylenetetrahydrofolate is a cofactor that participates in TS (thymidylate synthase)-mediated methylation of dUMP to generate dTMP. Previous studies have shown that TS is a transcription factor that impairs the translation of P53 mRNA [186]. As the basic component of nucleotides in proliferating cells, purine is associated with cancer progression. Phosphoribosyl aminoimidazole carboxylase and adenylosuccinate lyase have been identified as oncogenes involved in CRC development [180, 187]. MYC induces metabolic changes by accelerating the expression of nucleotide-producing metabolic genes, driving enhanced nucleotide synthesis for CRC growth [6].

## Tumor-specific metabolic factors as therapeutic targets

Multiple standard therapy options are applied to CRC, including surgical removal followed by adjuvant chemotherapy and immunotherapy [188]. Increasing attention to CRC metabolic reprogramming has provided novel targets for the development of drug therapeutics, and some of these therapies are being currently tested in clinical trials. Combination treatment with different drugs to target different enzymes in CRC circumvents the development of chemoresistance. Therefore, because of the targetable vulnerability of enzymes involved in CRC metabolic reprogramming, certain inhibitors have been investigated for cancer therapy (Table 1).

**Table 1:** Selected small molecules with potential for clinical translation in CRC

Pathway	Target	Representative drugs	Comments	Most advanced clinical phase	Trial ID
Glycolysis	GLUT1	BAY-876	Inhibitor	Preclinical	NA
		STF-31	Inhibitor	Preclinical	NA
	HK	2-Deoxy-D-glucose	Competitive HK1 inhibitor	Preclinical	NA
		Lonidamine	HK2 inhibitor	Preclinical	NA
		3BP	HK2 inhibitor	Preclinical	NA
	PFKFB3	PFK-158	Inhibitor	Phase 1	NCT02044861
	PKM2	Shikonin	Inhibitor	Preclinical	NA
		TEPP-46	Activator	Preclinical	NA
	LDHA	GNE-140	Inhibitor	Preclinical	NA
TCA cycle	PDH	CPI-613	PDK activator	Phase 1&2	NCT01832857 NCT02232152 NCT02232152
		DCA	PDK2 inhibitor	Phase 1	NCT00566410
		Vitamin C	PDK1 inhibitor	Phase 1&2	NCT04035096
	IDH	AG-221	IDH2 inhibitor	Phase 1&2	NCT02273739
		AG-120	IDH1 inhibitor	Phase 2	NCT04056910
Pentose phosphate pathway	G6PD	RRx-001	Inhibitor	Phase 2	NCT02096354
		G6PDi-1	Inhibitor	Preclinical	NA
	ME	NPD389	ME2 Inhibitor	Preclinical	NA
Glutamate and Glutamine metabolism	GLS	CB-839	Allosteric inhibitor	Phase 1&2	NCT02861300 NCT03965845 NCT03263429
	GPT	Aminooxyacetate	Pan-aminotransferase inhibitor	Preclinical	NA
	ASCT2	V-9302	Aantagonist of glutamine transporter	Preclinical	NA
	xCT	Sulfasalazine	Ferroptosis inducer	Phase 3	NCT01198145
SGOC metabolism	PHGDH	NCT-503	Non-competitive inhibitor	Preclinical	NA
	SHMT	SHIN1	Dual SHMT 1/2 inhibitor	Preclinical	NA
	MTHFD2	LY345899	Folate analogue	Preclinical	NA
Tryptophan metabolism	IDO1	1-L-MT	Competitive inhibitor	Phase 1	NCT05469490 NCT00739609
		Epacadostat	Specifical inhibitor	Phase 1&2	NCT02178722 NCT03361228 NCT02327078 NCT03516708 NCT03182894
	TDO	HTI-1090	Dual IDO1/TDO inhibitor	Phase 1	NCT03208959
	AhR	BAY2416964	AHR antagonist	Phase 1	NCT04069026 NCT04999202
		IK-175	AHR antagonist	Phase 1	NCT04200963
Fatty acid metabolism	ACLY	NDI-091143	Inhibitor	Preclinical	NA
	ACC	Soraphen A	Inhibitor	Preclinical	NA
	FASN	TVB-2640	Inhibitor	Phase 1	NCT02980029
	ACSL	Triacsin C	PUFA analog	Preclinical	NA
	SCD1	A939572	Inhibitor	Preclinical	NA
Cholesterol metabolism	CPT1	Perhexiline	Inhibitor	Preclinical	NA
	SREBPs	Fatostatin	SREBP activity inhibitor	Preclinical	NA
	ACAT1	CI-976	Competitive inhibitor	Preclinical	NA
	HMGCR	Simvastatin	Competitive inhibitor	Phase 2&3	NCT00994903 NCT02161822 NCT01238094 NCT02026583 NCT01110785 NCT00313859
		Rosuvastatin	Competitive inhibitor	Phase 1&2&3	NCT02569645 NCT01011478 NCT05368805
	LXR	LXR623	LXR agonist	Preclinical	NA
		GW3965	LXR agonist	Preclinical	NA
Nucleotide metabolism	DHODH	Leflunomide	Inhibitor	Phase 1	NCT04997993
	TS	5-fluorouracil	TS inhibitor	Phase 1&2&3&4	NCT02582970 NCT00932438 NCT00046995

### Targeting glucose metabolism

Targeting that increases aerobic glycolysis flux represents a promising treatment for CRC. Although GLUT1 is a potential target, no GLUT1 inhibitor has been used in the clinic. BAY-876 and STF-31 are selective GLUT1 inhibitors that prevent the uptake of glucose and, as a result, block carbon flux into glycolysis [189, 190]. The glucose analog 2-deoxy-d-glucose disrupts glucose metabolism by inhibiting GLUT1 and HK2, which can restore cell chemosensitivity to lovastatin [191]. Lonidamine and 3BP (3-bromopyruvate) both inhibit HK2. Lonidamine slows the development of CRC and induces immunogenic cell death through amplified immunotherapy [192]. Treatment with 3BP significantly suppresses KRAS^G13D^- and BRAF^V600E^-mutant tumor growth but shows fewer inhibitory effects on cells lacking mutant alleles [4]. PFK-158 suppresses PFKFB3. A preclinical study showed the great promise and safety of the PFKFB inhibitor PFK-158 [193]. It is unknown whether activating or inhibiting PKM is effective in the treatment of cancer because tumor cells show greater benefit from low PKM2enzyme activity. Shikonin, a natural compound in the traditional Chinese medicinal herbal medicine Zicao, impedes CRC cell proliferation via inhibition of PKM2 [194]. In turn, TEPP-46 induces high PKM2 tetramer activity, leading to the inhibition of its nuclear translocation [195]. GNE-140, a derivative of piperidine, exhibits a greater inhibitory effect on the enzymatic activity of LDHA and induces OXPHOS metabolic profile acquisition to attenuate tumor cell growth [196]. Inhibiting PDH and OGDH disrupts mitochondrial metabolism and exhibits widespread antitumor efficacy. Notably, CPI-613, a fatty acid analog, inhibited PDH and OGDH to slow cell proliferation [197]. DCA shows great antitumor activity and enhances chemotherapeutic effects in CRC via the P53/miR-149-3p/PDK2 axis [55]. Additionally, vitamin C stimulates PDH via the inhibition of PDK1 [198]. Although vitamin C administered in conjunction with first-line treatment failed to prolong PFS in CRC patients, ongoing phase 2 trials continue to shed light on the use of vitamin C in solid tumor treatment [199]. Both the FDA-approved IDH1^R132^ inhibitor AG-120 and the IDH2^R140^ and IDH2^R172^ inhibitor AG-221 are being evaluated in several clinical trials for the treatment of hematological malignancies. Except for its inhibitory effect on IDH1^R132^, AG-120 blocked ASCT2-mediated metabolic reprogramming in CRC cells, showing their growth [200]. RRx-001 is a G6PD inhibitor that lowers the ribonucleotide synthesis rate and GSH levels. Combination treatment with RRx-001 led to an improved clinical prognosis compared to that associated with currently approved chemotherapy [201]. G6PDi-1, a nonsteroidal agent, unexpectedly reduced the NADPH/NADP^+^ ratio by inhibiting G6PD [202]. ME2 produces NADPH to maintain redox balance and fatty acid biosynthesis. As a potential treatment, the ME2 inhibitor NPD389 has been evaluated in preclinical trials [203].

### Targeting amino acid metabolism

Undoubtedly, reprogrammed amino acid metabolism controls the availability of amino acids; therefore, targeting amino acid metabolism may lead to metabolic vulnerability in CRC cells. PIK3CA^p110α^-mutant cells were more sensitive to environmental glutamine, which indicates that it may be a vulnerable target in CRC [89]. The combination treatment of the GLS inhibitor CB-839 with capecitabine showed dramatically increased therapeutic efficiency in CRC cells with PIK3CA^p110α^ mutations in both preclinical and clinical trials [204]. Aminooxyacetate is a pan-aminotransferase inhibitor that potently repressed the growth of PIK3CA^p110α^ mutant xenograft CRC tumors, and the antitumor effect of aminooxyacetate on PIK3CA^p110α^-mutant xenograft tumors has been proven to be due to the inhibition of GPT2 [89]. In addition to the previously mentioned treatment AG120, V-9302 is another ASCT2 inhibitor, and V-9302 treatment abrogates cell growth and increases the cell death rate [205]. Sulfasalazine inhibits xCT and has been demonstrated to induce cancer cell apoptosis by preventing xCT system-dependent GSH production [206]. NCT-503, a PHGDH inhibitor, significantly attenuated tumor progression in CRC patient-derived xenograft tumors and suppressed tumor cell growth in a dose-dependent manner [108]. The development of dual SHMT1/2 inhibitors was aimed at blocking glycine metabolism. Through the prevention of glycine synthesis that results in the progressive depletion of purines, SHIN1 leads to the loss of nucleotide triphosphates and further blocks cell growth [119]. MTHFD2 endows CRC cells with greater capacity for malignancy, and its inhibitor LY345899 displays therapeutic activity and is thus a potential target for further clinical investigation [125]. In conventional therapies, tryptophan metabolic enzymes are common therapeutic targets that show great potential in cancer therapy; however, the results from translational clinical trials based on these enzymes have been frustratingly contradictory [131]. 1-L-MT (1-L-Methyltryptophan), a competitive IDO1 inhibitor, abrogates human CRC cell proliferation by inducing mitotic death [207]. The IDO1 inhibitor epacadostat is currently undergoing clinical investigation in various tumor types, including CRC [208]. Clinical trials for evaluating combinations of epacadostat and other antitumor drugs have been completed or are ongoing; these combinations include epacadostat with pembrolizumab and azacitidine (NCT03182894), or epacadostat with MK-3475 (NCT02178722). Furthermore, the dual IDO1/TDO inhibitor HTI-1090 has been entered into clinical trials of advanced solid tumors including CRC (NCT03208959). AhR, a cytosolic transcription factor, exerts key roles to regulate immunity and cell differentiation and has recently been shown to mediate several important functions by binding to certain tryptophan intermediates [131]. In advanced solid tumors, the AhR antagonists BAY2416964 and IK-175 specifically have been reported to bind AhR and subsequently inhibit AhR activation (NCT04069026 and NCT04200963).

### Targeting lipids metabolism

Deregulated lipid metabolism favors cancer cell growth, and lipogenic enzymes constitute viable targets for cancer treatment. ACLY forms an atomic structure with a hydrophobic cavity and can bind small-molecule inhibitors such as NDI-091143 [209]. To inhibit fatty acid synthesis, Soraphen A has been demonstrated to block ACC function to attenuate the development of CRC [154]. TVB-2640 is a promising FASN inhibitor for solid cancers, including CRC [210], and is the only inhibitor of FASN to be evaluated in CRC clinical trials (NCT02980029 and NCT02223247). Targeting the abnormal ACSL/SCD lipid metabolic network is a promising strategy. Compounds that have been designed to target ACSLs, such as Triacsin C exhibited competitive inhibition of ACSL1/4 activity [211]. Systemic administration of A939572 has been shown to enhance antitumor immune responses in CRC by repressing SCD1 activity [212]. CPT1 mediates lipids transport between the cytosol and mitochondria. Perhexiline, a CPT1/2 inhibitor, initially cures patients with angina and has recently been reported to be a CRC treatment. Patient-derived tumor xenograft models showed that the combined use of perhexiline and oxaliplatin was effective in shrinking CRC tumors, revealing the clinical potential of using perhexiline for cancer therapy [213]. Intriguingly, blocking the formation of the SCAP (SREBP cleavage-activating protein)-SREBP complex is an effective strategy for inhibiting SREBP activity. Notably, a specific inhibitor of SCAP, fatostatin, has been reported to bind to the SCAP protein and block the formation of the SCAP-SREBP complex, which inhibits SREBP activity and subsequently attenuates the expression of lipogenic genes and alters cellular metabolism, thereby controlling cancer cell survival [214]. Through microscopy, the structural details of ACAT1 have been revealed. The small-molecule inhibitor CI-976 interacts with ACAT1, which may provide information useful for developing ACAT1 inhibitors for the treatment of associated diseases [215]. Statins inhibit cholesterol synthesis by targeting HMGCR and are considered to constitute the gold standard of care for preventing CRC incidence and reducing the recurrence and risk of CRC-related risk [216]. LXR623 and GW3965 both cause LXR activation, which disrupts cholesterol homeostasis and predominantly increases caspase-dependent apoptosis [171, 174].

### Targeting nucleotide metabolism

Depleting nucleotide pools has long been regarded as a feasible option for CRC treatment. DHODH is usually targeted in the treatment of immune diseases and shows antitumor activity in CRC. The DHODH inhibitor leflunomide shrinks the progression of CRC in vivo [217]*.* Furthermore, the natural compound 3,3’-diindolylmethane has also been identified as an inhibitor of DHODH[218]. TS is the direct protein target of 5-fluorouracil. Mechanistically, 5-fluorouracil was catalyzed into fluorodeoxyuridine monophosphate, which forms a complex with TS that blocks the conversion of dTMP from dUMP[219].

## Conclusions and remarks

Tumor metabolism reprogramming has emerged as a hallmark and important therapeutic target for solid tumors, including CRC. Similar to other landmark findings, research continues to provide insight into the role played by tumor metabolism reprogramming in CRC, but numerous questions are as remain to be clarified. (1) Notably, all rate-limiting enzymes have been identified in the context of red blood cells, and the characterization of rate-limiting enzymes is not fixed. Macrophages promote tumor cell growth and are dependent on PGK1-mediated aerobic glycolysis, which changes PGK1 into a limiting enzyme in cancer cells [220]. Interestingly, GAPDH was surprisingly identified via metabolomic research as a potentially rate-limiting enzyme [221]. (2) The functional diversity of genes is a confounding factor. Although upregulated PKM2 is a common feature that is thought to promote tumorigenesis, a study showed that, in contrast, PKM1 but not PKM2 exhibited a tumor-promoting function [222, 223]. Furthermore, PKM2 is a phosphatase that regulates protein substrates in an epigenetic manner. (3) Due to the plasticity of the metabolic pathway and the heterogeneity of tumors, few drugs have been approved for clinical use. P53 and TAp73 regulate G6PD activity to control glucose flux into the PPP pathway during CRC progression, while KRAS-mutant cancer cells show no G6PD dependence [8, 74, 75]. The results of clinical trials performed to evaluate promising IDO1 inhibitors have been disappointing, which highlights the need for a deeper understanding of the precise effector mechanisms of tumor metabolism in vivo [131]. Additionally, only 13% of CRC cases have been characterized by metabolic reprogramming (CMS3) [224, 225].

Enzymes involved in metabolic reprogramming are altered to fuel CRC carcinogenesis and resistance to stress. Targeting metabolic enzymes has transformed the treatment landscape and outcomes for cancer patients. Alteration of metabolic enzymes is common in CRC and further understanding of the precise downstream effector mechanisms of colorectal cancer metabolism reprogramming will be indispensable for drug development.

## Data Availability

Not applicable.

## Abbreviations

CRC: Colorectal cancer
ATP: Adenosine triphosphate
TCA: Trichloroacetic acid
OXPHOS: Oxidative phosphorylation
PPP: Pentose phosphate pathway
NADPH: Nicotinamide adenine dinucleotide phosphate
NADH: Nicotinamide adenine dinucleotide
GLUT: Glucose transporter
ERK: Extracellular regulated kinase
CSN6: COP9 signalosome subunit 6
PKC: Protein kinase C
ROS: Reactive oxygen species
GAPDH: Glyceraldehyde-3-phosphate dehydrogenase
mTOR: Mammalian target of rapamycin
YAP: Yes-associated protein
JMJD2B: Jumonji domain-containing protein 2B
AMPK: Adenine monophosphate-activated protein kinase
CREB1: CAMP response element binding protein 1
HK: Hexokinase
TIGAR: TP53 inducible glycolysis and apoptosis regulator
PITA: P53 inhibitor of TIGAR activation
G6P: Glucose-6-phosphate
Skp2: S-phase kinase-associated protein 2
PLK3: Polo-like kinase 3
STAT3: Signal transducer and activator of transcription 3
PFK1: Phosphofructokinase-1
PFKFB: 6-Phosphofructo-2-kinase/fructose-2,6-bisphosphatase
CO/CBS: Carbon monoxide/cystathionine β-synthase
PKM: Pyruvate kinase M
HIF1α: Hypoxia-inducible factor 1 subunit α
LDHA: Lactate dehydrogenase A
PGC1α: PPARγ coactivator 1α
PDH: Pyruvate dehydrogenase
OGDH: Oxoglutarate dehydrogenase
PDK1: Pyruvate dehydrogenase kinase 1
PGK1: Phosphoglycerate kinase 1
DCA: Dichloroacetate
IDH: Isocitrate dehydrogenase
α-KG: α-Ketoglutarate
SIRTs: Sirtuins
R-2HG: R-2-hydroxyglutarate
ME: Malic enzyme
GSH: Glutathione
G6PD: 6-Phosphogluconate dehydrogenase
PGLS: 6-Phosphogluconolactonase
6PGD: 6-Phosphogluconate dehydrogenase
LKB1: Liver kinase B1
PP2A: Protein phosphatase 2A
TKT: Transketolase
RPIA: Ribose-5-phosphate isomerase A
PRPS1: Ribose-phosphate diphosphokinase 1
GLS: Glutaminases
GPT: Glutamate pyruvate transaminase
GDH: Glutamate dehydrogenase
GOT: Glutamate oxaloacetate transaminase
GCN2: General control non-depressible 2
SOX12: SRY-box transcription factor 12
HSF1: Heat shock factor 1
AhR: Aryl hydrocarbon receptor
CEMIP: Cell migration-inducing and hyaluronan-binding protein
SSP: Serine synthesis pathway
SGOC: Serine-glycine-one-carbon
PHGDH: Phosphoglycerate dehydrogenase
PSAT: Phosphoserine aminotransferase
ILF3: Interleukin enhancer-binding factor 3
SHMT: Serine hydroxymethyltransferase
MTHFD: Methylene-tetrahydrofolate dehydrogenase
TPH1: Tryptophan hydroxylase 1
IDO1: Indoleamine 2,3-dioxygenase 1
LDs: Lipid droplets
FAO: Fatty acid oxidation
ACLY: ATP citrate lyase
ACC: Acetyl-CoA carboxylase
PPARδ: Peroxisome proliferator-activated receptor δ
FASN: Fatty acid synthase
ACSL: Acyl‐CoA synthetase
SCD: Stearoyl-CoA desaturase
CPT1: Carnitine palmitoyltransferase 1
SREBPs: Sterol regulatory element-binding proteins
ACAT1: Acetyl-CoA acetyltransferase 1
HMGCR: Hydroxymethylglutaryl-CoA reductase
LDLR: Low-density lipoprotein receptor
LXR: Liver X receptor
ABCA1: ATP-binding cassette transporter A1
USP20: Ubiquitin-specific peptidase 20
FDFT1: Farnesyl-diphosphate farnesyltransferase 1
PRPS1: Ribose-phosphate diphosphokinase 1
CAD: Carbamoyl phosphate synthetase/aspartate transcarbamylase/dihydroorotase
DHODH: Dihydroorotate dehydrogenase
TS: Thymidylate synthase
3BP: 3-Bromopyruvate
1-L-MT: 1-L-methyltryptophan
SCAP: SREBP cleavage-activating protein
